# Comparison of Plasmodium Vivax Infections in Duffy Negatives From Community and Health Center Collections in Ethiopia

**DOI:** 10.21203/rs.3.rs-3385916/v1

**Published:** 2023-10-03

**Authors:** Lauren Bradley, Delenasaw Yewhalaw, Elizabeth Hemming-Schroeder, Brook Jeang, Ming-Chieh Lee, Endalew Zemene, Teshome Degefa, Eugenia Lo, Christopher King, James Kazura, Guiyun Yan

**Affiliations:** University of California Irvine; Jimma University; Colorado State University; University of California Irvine; University of California Irvine; Jimma University; Jimma University; Drexel University; Case Western Reserve University; Case Western Reserve University; University of California Irvine

**Keywords:** Malaria, Duffy blood group, Plasmodium vivax, qPCR, gene copy number

## Abstract

**Background:**

Malaria remains a significant cause of morbidity and mortality in Ethiopia with an estimated 4.2 million annual cases and 61% of the population living in areas at risk of malaria transmission. Throughout the country *Plasmodium vivax* and *P. falciparum* are co-endemic, and Duffy expression is highly heterogeneous. The public health significance of Duffy negativity in relation to *P. vivax* malaria in Ethiopia, however, remains unclear.

**Methods:**

A total of 9,580 and 4,667 subjects from community and health facilities from a malaria endemic site and an epidemic-prone site in western Ethiopia were enrolled and examined for *P. vivax* infection and Duffy expression. Association between Duffy expression, *P. vivax* and *P. falciparum* infections were examined for samples collected from asymptomatic community volunteers and symptomatic subjects from health centers.

**Results:**

Among the community-based cross-sectional samples, infection rate of *P. vivax* among the Duffy positives was 2–22 fold higher than among the Duffy negatives. Parasite positivity rate was 10–50 fold higher in Duffy positive than Duffy negatives among samples collected from the health center settings and mixed *P. vivax* and *P. falciparum* infections were significantly more common than *P. vivax* mono infections among Duffy negative individuals. *P. vivax* parasitemia measured by 18sRNA parasite gene copy number was similar between Duffy positives and Duffy negatives.

**Conclusions:**

Duffy negativity does not offer complete protection against infection by *P. vivax*, and cases of *P. vivax* in Duffy negatives are widespread in Ethiopia, being found in asymptomatic volunteers from communities and in febrile patients from health centers. These findings offer evidence for consideration when developing control and intervention strategies in areas of endemic *P. vivax* and Duffy heterogeneity.

## Background

Despite significant progress towards malaria control in the past two decades, malaria remains a major cause of mortality and morbidity in Africa^1^. According to the World Health Organization, *Plasmodium vivax* and *P. falciparum* contributed to approximately 700 thousand and 230 million cases, respectively, in Africa in 2021^2^. Current endemicity of *P. vivax* in Africa correlates with areas of high heterogeneity in Duffy expression^3,4^. The Duffy antigen receptor for chemokines (DARC), often referred to as the Fy glycoprotein, is a silent heptahelical chemokine receptor located on chromosome 1 and expressed on the surface of erythrocytes. DARC has been recognized as the binding antigen of *P. vivax*, and a single point mutation located in the GATA-1 transcription factor binding site of the DARC gene promoter (−67T > C) causes this receptor to not be expressed, resulting in a Duffy negative phenotype^5,6^. The absence of this receptor on red blood cells has been shown to confer resistance to blood-stage infection by *P. vivax*^3, 7, 8^. Despite this established dogma, cases of *P. vivax* are being found in confirmed Duffy negative individuals throughout different African countries^9-13^. Whether discovery of increasing number of *P. vivax* infections in Duffy negatives results from more recent research on *P. vivax* in Africa or from new *P. vivax* genetic variants, the data suggest that Duffy negativity no longer confers complete resistance to blood-stage *P. vivax* infection^14, 15^.

There is little information on the public health significance of *P. vivax* infection in individuals lacking the Duffy antigen in Africa. For example, how frequent are Duffy negative individuals infected with *P. vivax* compared to Duffy positive individuals from the same communities? How frequently does *P. vivax* contribute to clinical malaria among Duffy negatives compared to Duffy positives from areas of same endemicities? This population-based study aimed to address these questions in two locations with varying malaria endemicities in southwestern Ethiopia, using samples from communities and health centers. Despite significant progress towards malaria control in the past two decades, malaria remains a major cause of mortality and morbidity in Ethiopia, with an estimated annual 4.2 million annual cases^2, 16^. *Plasmodium vivax* and *P. falciparum* accounted for approximately 33% and 67% of all malaria cases, respectively, and it is one of only a few countries in Africa where *P. vivax* remains consistently endemic^4^.

## Methods

### Study Sites

**Figure 1:**
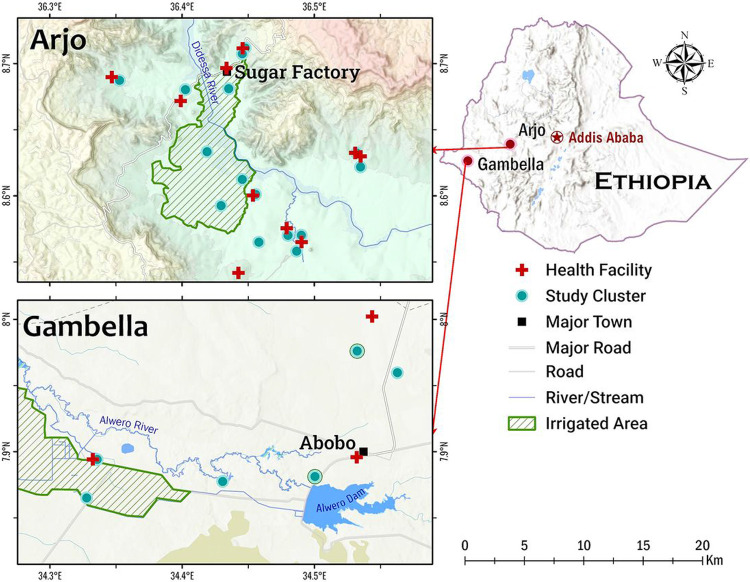
Map showing location of both study sites; Arjo and Gambella, in western Ethiopia. Includes locations of study clusters, health facilities, and major towns in the regions.

Samples were collected from two study sites, Arjo-Didessa and Gambella, both located in western Ethiopia (Fig. 1) with a rainy season lasting from May to October. The Arjo-Didessa sugarcane plantation is located within the Oromia Regional State 395 km west of the Ethiopian capital Addis Ababa and the area covers most of the Arjo-Didessa sugarcane irrigation scheme. It sits at an elevation ranging from 1200m to 1500m above sea level, and comprises 15 villages in 3 districts (Jimma Arjo, Bedele District, and Dado Hana District). It contains 1 health center, 3 health posts, and 9 command posts which are smaller scale health posts located within the temporary residential areas formed by migrant workers. The sugarcane plantation was formerly the Didessa Wildlife Sanctuary before 2006 when the state owned sugarcane plantation was developed to supply the proximal sugarcane factory. It is one of the biggest sugarcane developments in the country, currently covering 5000 hectares with plans to expand to 80,000 hectares^17-19^. Gambella is located in the Abobo District in the Gambella Regional State, 811 km west of Addis Ababa. The area’s elevation ranges from 400-600m above sea level and as of 2019 had a population of 20,080. The main socioeconomic activity in the area is farming of cotton, maize and sorghum, or working fruit plantations to produce mango, papaya and banana. Additionally, the Alwero Dam provides fishing opportunities and employs approximately 2000 people at a large-scale rice irrigation scheme that currently spans 3,000 hectares with plans to expand to 10,000 hectares. The district comprises 19 villages containing 4 health centers and 16 health posts^20, 21^. These sites were chosen for the study as both areas have high levels of Duffy admixture, and continuous *P. vivax* endemicity^11, 22^.

### Blood sample collection

Finger prick blood samples were collected throughout both study sites from asymptomatic community members during cross-sectional surveys and febrile patients at health centers. From each individual, a total of 3 blood spots, equaling ~ 50ul, was pressed to Whatman 3MM filter paper for storage and transportation. For community collections all residents willing to participate were included in the study and provided signed informed consent and/or assent for minors under 18 years old. At the time of sample collection, for both clinical and community samples, the age and sex of participants were recorded when possible. Dried blood spots were transported to the University of California Irvine and stored at 4C.

### DNA Extraction and qPCR

Parasite DNA was extracted from dried blood spots (DBS) using a standardized saponin/chelex method^23^. DNA was eluted to ~ 200ul molecular grade water stored at 4C in the short term or −20C for long term storage. *Plasmodium* species-specific primers and probes were used to amplify the 18sRNA gene using a previously described protocol with modification^24^. Real time PCR was conducted at a total volume of 12ul containing; 6μl ThermoFisher FastAdvanced MM (2X), 0.5μl of each species-specific probe, 0.4μl of each forward and reverse species-specific primer, and 2μl parasite DNA. Reaction conditions were set as follows: 50C for 2 min, and 45 cycles of 95C for 2 min, 95C for 3 seconds, 60C for 30 seconds and run on a QuantStudio 3 Real-Time PCR System.

### Duffy Sequencing

An approximately ~ 600-bp fragment of the human DARC gene encompassing the – 33rd nucleotide position located in the GATA-1 box of the promoter region was amplified sequenced following established protocols to assess Duffy expression^22, 25, 26^. Specifically, the total volume for amplification was a 20ul reaction mixture containing; 10μl DreamTaq Green PCR MM (2X), 0.3μl of each forward and reverse primer, and 2μl genomic DNA. Thermocycling conditions were set at; 94C for 2 minutes, 35 cycles of 94C for 30 seconds, 61 for 30 seconds, and 65 for 40 seconds followed by a 2-minute extension at 65C. Five microliters of PCR product were run on a 1.5% agarose gel to confirm amplification. PCR product which had successful amplification was cleaned was cleaned enzymatically to remove remaining primers and dNTPs; 2μl SAP and 0.2μl XO1 per was added to PCR product and cleaned via the following thermocycling conditions; 37C for 15 minutes, 80C for 15 minutes, and then held at a 4C extension. Sanger sequencing was conducted by Retrogen Inc. using forward primers and chromatogram results were visually analyzed via Chromas for a T◊C mutation at the 33rd nucleotide position indicating Duffy negativity. Only samples positive for *P. vivax* were sequenced for Duffy expression.

### Data Analysis

Malaria prevalence was calculated for both study settings at each study site separately. Overall *Plasmodium* prevalence was compared between study sites for community and clinical collections via the Chi-Square test for independence. Given that only *P. vivax* positive samples were sequenced for Duffy expression, rates of Duffy negativity in the population was not directly assessed for this study, the overall rate of *P. vivax* in Duffy negative and Duffy positive individuals was calculated by dividing the number of *P. vivax* infections by the expected number of Duffy negative and positive individuals at each site. Expected Duffy negative and positive populations were calculated by multiplying the total number of samples by the Duffy negativity rate in Arjo (43.6%) and Gambella (45.9%) as determined in a previously published study^27^. The ratio of mixed (Pv + Pf) to mono (Pv) infections was determined for each study setting, community and health facility, for both Duffy negatives and Duffy positives. Comparisons of the rate of *P. vivax* in Duffy negatives to Duffy positives, and the and the ratio of mixed to mono infections for Duffy negatives and positives were made via Fisher’s Exact test for both community and health facility collected samples. Parasite Gene Copy Number (GCN) was calculated from qPCR Cycle threshold (Ct) values via standard curve to estimate parasite density. Log_10_ transformed GCN was compared between community and health facility settings for both *P. vivax* and *P. falciparum* via two-sample t-test, and between Duffy negatives and Duffy positives for both settings via Fisher’s Exact test.

## Results

### Prevalence of PV Across Study Sites, Collection Method and Duffy Expression

A total of 14,247 dried blood spots were collected from two study sites in southwestern Ethiopia (Fig. 1) from February 2018 to December 2021. Asymptomatic community collections were made via cross-sectional surveys conducted during the spring and late-fall of each year and making up 9,580 of the total dried blood spots. The remaining 4,667 samples were symptomatic infections collected from health clinics and facilities in the regions via passive case detection (PCD). In total 344 DBS were positive for only *P. vivax*, 937 for only *P. falciparum* and 35 samples exhibited a mixed infection being positive for both *P. vivax* and *P. falciparum* (Table 1). A total of 7,519 of these DBS were collected from Arjo; 5,454 from cross-sectional surveys and 2,065 from passive case detection. In Gambella 6,728 samples were collected in total; 4,126 were collected from the community during cross-sectional surveys and 2,602 via passive case detection (Table 1). Overall, *P. vivax* and *P. falciparum* infection rate was significantly higher in Gambella than in Arjo (P < 0.001 for both species).

Duffy genotyping was performed only on *P. vivax* mono infections and mixed *P. vivax* and *P. falciparum* infections across all study sites and collection methods (Table 2). Of the 379 *P. vivax* positive and mixed-species infections, 345 were successfully sequenced at the T33C promoter of the GATA-1 transcription factor. Among the community-based cross-sectional samples, infection rate of *P. vivax* among the Duffy negatives and positives was low and similar in Arjo, but significantly higher infection rate was found in Gambella among Duffy positives than Duffy negatives (5.6% vs. 0.26%, P < 0.001; Table 2). Similarly, sample positivity rate was more than 10–50 fold higher in Duffy positive than Duffy negatives in both sites among samples collected from the health center settings (Table 2), suggesting a much reduced *P. vivax* burden among Duffy negative people in febrile patients.

Interestingly, we found that a considerably large proportion of malaria infections were mixed species infection. Among the community-based samples, eight out of 133 (6.0%) malaria infections were mixed species and 26 out of 212 (12.3%) samples were mixed infections from the health center settings (Table 3). Among the Duffy negatives, *P. vivax* was found more frequently found in the form of mixed-species infection than mono infections, whereas mono *P. vivax* infections were far more common in Duffy positives. In the community asymptomatic samples, the ratio of mixed species infection to *P. vivax* mono infection was 0.5 among Duffy negatives, but this ratio was reduced to 0.05 in Duffy positives (P < 0.05; Table 3). In febrile samples from health centers the ratio of mixed species infection to *P. vivax* mono infection was 3.5 among Duffy negatives, far greater than the ratio observed in Duffy positive (0.10; P < 0.001). This data strongly suggests that in Duffy negative individuals *P. vivax* is more frequently found in mixed infections compared to *P. vivax* only mono infections.

### P. vivax Parasitemia in Community and Clinical Samples and Across Duffy Expressions

**Figure 2:**
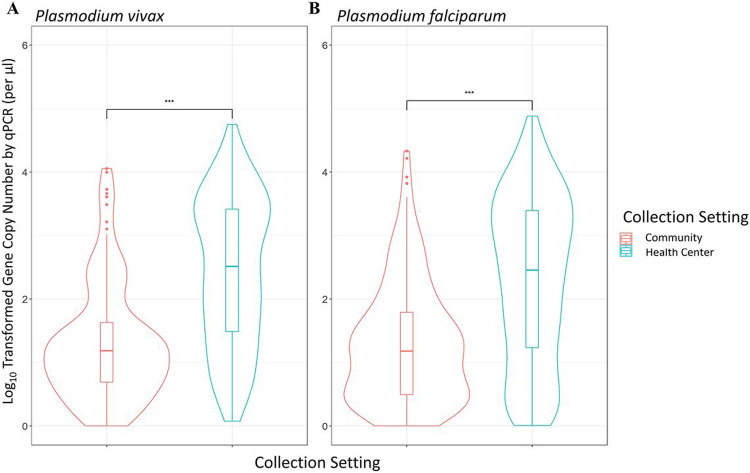
Log-transformed parasite gene copy number of community and clinical samples. Violin box-plot of the log-transformed parasite Gene copy number (GCN) of **(A) *Plasmodium vivax*** and **(B) *Plasmodium falciparum*** by qPCR for individuals of all ages. These samples were collected both in the community via cross-sectional surveys and at health centers via Passive Case Detection (PCD) at two Ethiopian field sites; Arjo and Gambella. The central box represents the interquartile range with the median shown as the center line in the box. P-values (above) are calculated via two-sample T.test (***, P < 0.001).

**Figure 3:**
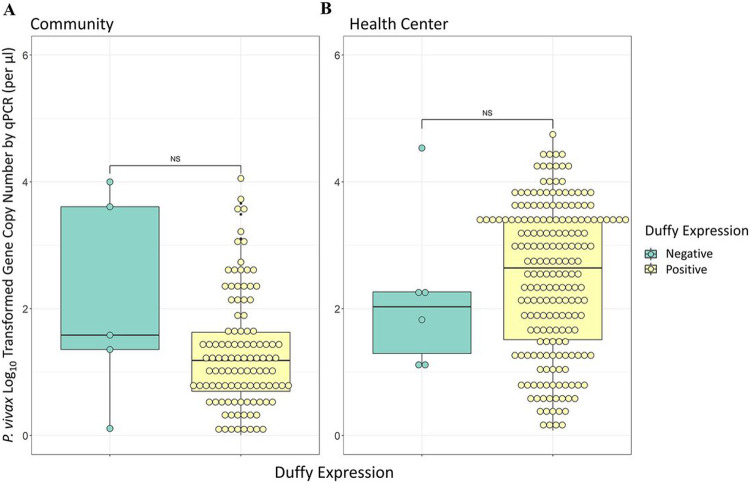
Box plots of the log-transformed parasite gene copy number for Duffy negative and Duffy positive individuals. Box plot of the log-transformed gene copy number for *Plasmodium vivax* for Duffy negative and Duffy positive individuals of all ages in both **(A)** the community via cross-sectional survey and **(B)** health centers via Passive Case Detection. Box plots represent the interquartile range with the median expressed as the center line. All individual data points are shown via open circles, P-values were calculated via Fisher’s Exact test (NS, non-significant).

Analyses of qPCR data revealed significant differences in the parasitemia between cross-sectional samples without clinical symptoms and clinical samples collected during passive case detection from health centers for both *P. vivax* and *P. falciparum*. In both *P. vivax* and *P. falciparum* infections parasitemia was significantly higher in samples collected via passive case detection than via cross-sectional survey (P < 0.001, Fig. 2). Symptomatic *P. vivax* infections showed a geometric mean gene copy number (GCN) of 2.03 parasites/μl, which was significantly higher than the asymptomatic *P. vivax* infections, which had a geometric mean of 0.94 parasites/μl (P < 0.001, Fig. 2). Similarly, symptomatic *P. falciparum* infections exhibited a geometric mean of 1.67 parasites/μl, which was significantly higher than the asymptomatic *P. falciparum* infections which had a mean of 0.90 parasites/μl (P < 0.001). Community Duffy-negative and Duffy-positive samples exhibited a similar parasitemia, with a GCN of 1.28 and 0.93 parasites/μl respectively (P > 0.05, Fig. 3). Similarly, PCD Duffy-negative and Duffy positive samples showed a mean GCN of 1.93 and 2.07 parasites/μl respectively (P > 0.05, Fig. 3). These data do not include four Duffy negative samples as their gene copy numbers fell just outside of our standard curve based cutoff range. Given the substantial differences in sample sizes between Duffy-negatives and Duffy-positives it is possible that the lack of significance observed here is indeed due to a small samples size of Duffy negatives.

## Discussion

This study sought to examine *P. vivax* malaria burden in Duffy negative individuals at two field sites with similar proportion of Duffy negativity, but different malaria endemicities in southwest Ethiopia. We found, firstly, that *P. vivax* posed a significant health burden at both sites, but was far more prevalent in the community in Gambella than in Arjo where infection prevalence was over 50 times higher. In febrile patients *P. vivax* was found more often in Arjo than in Gambella; however, this difference was much less drastic than in the community with Arjo exhibiting only 1.5 times more *P. vivax* clinical infections than Gambella. Across both sites and collection settings *P. vivax* was found far less frequently in Duffy negatives than Duffy positives. In the community Duffy positives had approximately 2 and 22-fold greater infection rate of *P. vivax* than Duffy negatives at Arjo and Gambella, respectively. In febrile patients and samples collected from health facilities this trend was even more apparent; in Arjo and Gambella Duffy positives exhibited a 51 and 10-fold greater positivity rate of *P. vivax* infections, respectively, than Duffy negatives. The variations in rate of infection were highly significant for samples from health centers at both sites, but only significant for community samples from Gambella. The lack of significance in Arjo community samples could potentially be due to the small sample size as only three *P. vivax* infections were found in the community in Arjo. These strongly suggest that *P. vivax* infections, despite being commonly found in Duffy negative individuals, are still predominantly occurring in Duffy positive people. Despite the significant variations in rate of *P. vivax* infection between Duffy expressions, we did not observe significant differences in parasitemia between Duffy negatives and Duffy positives in either the community or health centers. Perhaps most interestingly this study highlights a pattern of mixed versus mono infections related to Duffy negativity. By calculating the ratio of mixed to mono *P. vivax* infections among Duffy negative and positive individuals we found that Duffy negatives exhibited a 10 and 35-fold greater ratio of mixed to mono infections than Duffy positives in both the community and clinical settings, respectively. Therefore, for Duffy negatives, *P. vivax* is predominantly found in mixed infections more than mono infections.

Collectively our findings build on previous work documenting *P. vivax* infections in Duffy negative individuals in numerous African countries^28, 29^ including Cameroon^30^, Madagascar^9^, Angola and Equatorial Guinea^31^, Kenya^32^, Ethiopia^4^. These studies are consistent with our findings and support the conclusion that Duffy negative individuals are not completely resistant to infection by *P. vivax*, yet still have a greatly reduced prevalence of *P. vivax* infections compared to Duffy positive individuals. Our data show that despite no significant variation in parasitemia between Duffy positives and Duffy negatives, several *P. vivax* infections from Duffy negatives exhibited relatively high levels of parasitemia potentially implying that these parasites readily infect and adapt to Duffy negativity, allowing for greater erythrocyte invasion. Despite this, several studies have ample evidence that parasitemia of *P. vivax* is greatly reduced in Duffy negatives, supporting the hypothesis that parasite infectivity to the human erythrocyte, though not completely inhibited, is indeed reduced in the absence of the Duffy antigen^33, 34^. Several prior studies have also observed that *P. vivax* infections within Duffy negative individuals are frequently mixed infections, yet these data are limited in that they are predominantly descriptive and do not explore these mixed infections in detail nor compare their prevalence between Duffy negatives and positives^9, 35, 36^. Thus this current study stands out in its efforts to systematically evaluate the prevalence of mixed infections in individuals with Duffy negative status as compared to those with Duffy positive status. Our findings thus shed light on the noteworthy phenomenon that *P. vivax* infections in Duffy negatives frequently encompass mixed-species infections, especially when compared to Duffy positives.

Since the level of *P. vivax* exposure remained consistent among both Duffy positive and Duffy negative individuals across our distinct study locations, the observed diminished burden of *P. vivax* in Duffy negative individuals underscores that while Duffy negativity does not confer absolute resistance to *P. vivax* infection, it does exert a significant inhibitory effect on infection establishment. The mechanism behind *P. vivax* infections of Duffy negatives remains highly elusive, however, several studies have highlighted potential invasion mechanism adaptations of *P. vivax* that may circumvent Duffy-based infection inhibition and allow for infection on a lesser scale. One of the most well studied of these potential adaptations is the *P. vivax* Duffy binding protein 1 (PvDBPI) copy number expansion. Several different studies have clearly shown that PvDBP gene amplification both facilitated binding to alternative lower affinity receptors in Duffy negatives, and also suggested that the binding affinity of DARC with high copies of PvDBP could be much higher than with single-copy PvDBP parasites^14, 37-40^, providing a potential selective pressure towards gene duplication and thus increased infectivity. Two additional ligands, *P. vivax* glycosylphosphatidylinositol-anchored micronemal antigen (PvGAMA) and *P. vivax* merozoite surface protein-1 paralog (PvMSP1P), were recently found capable of binding to both Duffy positive and negative red blood cells, suggesting possible involvement in Duffy-independent invasion pathways^41^.

It warrants mention that in the present study is limited in that Duffy expression (negative vs. positive) was inferred based on genotype data of the T33C point mutation in the promoter region of the GATA-1 transcription factor binding site of the Duffy antigen receptor for chemokines (DARC) gene, which is known to alter erythroid expression and eliminate Duffy antigen expression on the red blood cell surface^25, 26, 42^. However, the direct antigen expression (phenotype) was not assessed. It is therefore not impossible for a genotypically categorized Duffy negative individual to potentially express Duffy receptors in some quantity, and the *P. vivax* strains infecting Duffy negatives in this study may be utilizing such an expression in invasion, despite determined genotypic negativity. Additionally, we did not assess the prevalence or burden of *P. falciparum* across Duffy negatives and positives as Duffy expression is not known to be associated with *P. falciparum* infection.

Understanding the distribution of *P. vivax* in Africa and exploring the significance of Duffy expression continues to be a challenging and intricate endeavor. Given the low parasitemia often associated with *P. vivax* infections of Duffy negative individuals, microscopy and RDTs are often not sensitive enough to detect infection, hindering their diagnosis and study in the field. Indeed, corresponding microscopy data from this area accounted for only approximately 70%, of all qPCR confirmed *P. vivax* positive infections^17^, highlighting the need for more sensitive molecular detection tools in the field. This has significant implications for malaria elimination on the continent as a high proportion of *P. vivax* cases are likely being overlooked by traditional diagnostic methods and thus going unaddressed in intervention and elimination efforts. It is clear through the current work that Duffy negativity is not a definitive barrier to infection, mixed infections are common in Duffy negatives, and that not only does *P. vivax* transmission remain widespread in Ethiopia, but asymptomatic community infections make up a significant portion of *P. vivax* cases resulting in a large undetected parasite reservoir that may greatly complicate and hinder interventions and elimination efforts. This information is vital to informing control and intervention strategies in areas of Sub-Saharan Africa with endemic *P. vivax* and areas of high Duffy heterogeneity.

## Figures and Tables

**Table 1:** PCR Prevalence of Plasmodium vivax (Pv) and P. falciparum (Pf) infections among community-based asymptomatic sampling and sample positivity among febrile patients detected by passive case surveillance from health centers in two sites in Ethiopia.

Settings	Site	Samples (n)	Total *Plasmodium* infections	Mixed Pv and Pf infections	Pv mono infections	Pf mono infections	P- value*
Community	Arjo	5454	19 (0.35%)	0	3 (0.06%)	16 (0.29%)	< 0.05
	Gambella	4126	424 (10.28%)	8 (0.19%)	133 (3.22%)	283 (6.86%)	< 0.001
	Total	9580	443 (4.62%)	8 (0.08%)	136 (1.42%)	299 (3.12%)	
Health Center	Arjo	2065	313 (15.16%)	9 (4.36%)	114 (5.52%)	190 (9.20%)	< 0.001
	Gambella	2602	560 (21.52%)	18 (0.69%)	94 (3.61%)	448 (17.22%)	< 0.001
	Total	4667	873 (18.79%)	27 (0.57%)	208 (4.46%)	638 (13.67%)	

* Fishers exact test comparison between Pv and Pf mono infection rate

**Table 2:** Rate of Plasmodium vivax (Pv) infections among Duffy negative and Duffy positive individuals in both community-based asymptomatic and passive case surveillance from health centers at two study sites in Ethiopia.

Setting	Site	Total Samples	Expected Duffy Negatives	Expected Duffy Positives	Total Pv Infections	Rate of Pv in Duffy Negatives	Rate of Pv in Duffy Positives	P- value*
Community	Arjo	5454	2525	2929	3	0.04% (1/2525)	0.07% (2/2929)	> 0.05
	Gambella	4126	1894	2232	130	0.26% (5/1894)	5.60% (125/2232)	< 0.001
Health Center	Arjo	2065	956	1109	120	0.21% (2/956)	10.64% (118/1109)	< 0.001
	Gambella	2602	1194	1408	92	0.59% (7/1194)	6.04% (85/1408)	< 0.001

* P-value calculated via Fishers Exact Test for comparing Rate of Pv in Duffy Negatives to Duffy Positives

**Table 3:** Distribution of Duffy phenotypes across Plasmodium vivax (Pv) and Mixed (Pv and Pf) infections in both community-based asymptomatic and passive case surveillance via health centers from two study sites in Ethiopia

Setting	Infection	n	Duffy negative	Duffy positive	Ratio of mixed species infection to *P.* *vivax* only infections		Fisher’s exact test
					Duffy negative	Duffy positive	
Community	Pv	125	4	121	0.5	0.05	P < 0.05
	Pv + Pf	8	2	6			
Health Center	Pv	186	2	184	3.5	0.10	P < 0.001
	Pv + Pf	26	7	19			
